# Pharmacokinetics of Macrolide Antibiotics and Transport into the Interstitial Fluid: Comparison among Erythromycin, Clarithromycin, and Azithromycin

**DOI:** 10.3390/antibiotics9040199

**Published:** 2020-04-22

**Authors:** Shinji Kobuchi, Teruhiko Kabata, Koki Maeda, Yukako Ito, Toshiyuki Sakaeda

**Affiliations:** Department of Pharmacokinetics, Kyoto Pharmaceutical University, Kyoto 607-8414, Japan; kobuchi@mb.kyoto-phu.ac.jp (S.K.);

**Keywords:** macrolide, pharmacokinetics, interstitial fluid, PK-PD

## Abstract

Recent research has found higher levels and longer total exposure of azithromycin, a macrolide antibiotic agent, in the interstitial fluid of the skin than in the plasma. This unique distribution is expected to contribute to its antimicrobial activity at the primary infection site. However, it remains unclear whether this characteristic distribution in the extracellular tissue space is common to macrolide antibiotics or if it is azithromycin-specific, with most macrolides largely localized intracellularly. In this study, we investigated pharmacokinetic characteristics of erythromycin and clarithromycin in the interstitial fluid of the skin of rats after intravenous drug administration, and compared the results with our previously reported results on azithromycin. Interstitial fluid samples were directly collected from a pore on the skin using a dissolving microneedle array. We found that the total macrolide concentrations in the interstitial fluid were significantly different among three macrolides. The rank order of the interstitial fluid-plasma concentration ratio was azithromycin (3.8 to 4.9) > clarithromycin (1.2 to 1.5) > erythromycin (0.27 to 0.39), and this ratio was stable after dosing, whereas higher drug levels in the skin tissue than in the plasma were observed for all three macrolides. Our results suggest that lower erythromycin concentrations in the interstitial fluid than in the plasma contributes to the emergence of bacterial resistance in the extracellular tissue space. Monitoring of total macrolide concentrations in interstitial fluid may provide valuable information regarding antimicrobial effects and the emergence of bacterial resistance for the development of an appropriate pharmacokinetics–pharmacodynamics-based dosing strategy.

## 1. Introduction

Antimicrobial resistance (AMR) results from the widespread use of antimicrobial agents and can endanger public health [1]. To prevent AMR and improve the clinical outcomes of antimicrobial agents, pharmacokinetic–pharmacodynamic (PK–PD)-based proper dose selection and treatment planning are required [2,3]. The interstitium is an important infection site for most bacteria [4,5], and the concentration of antimicrobial agents in the interstitial fluid (ISF), i.e., the extracellular tissue space fluid, is key to determine the pharmacological effects and emergence of bacterial resistance. However, most previous studies have noted the drug concentrations in the intracellular tissue space in vitro, such as in phagocytes [6,7], and in vivo using tissue homogenates [8,9], where the concentration represents a mixture of the intracellular and ISF contents, due to the relatively simple experimental procedures required. A limitation of these methods is that they do not estimate drug concentrations in the ISF. The microdialysis method can obtain an ISF sample [4,9], but this method may lead to inaccurate results because the sample is obtained by tissue perfusion; therefore, drug concentrations in the collected sample may represent the protein-unbound drug concentrations. Information regarding the unbound drug concentration does not help us to understand the transport of drugs into the extravascular space.

Recently, we successfully obtained ISF samples directly from a pore on the skin using dissolving microneedles (DMNs) [10,11] and determined the total concentrations of azithromycin (AZM), a 15-membered ring macrolide antibiotic agent, in the ISF of the skin of rats [12]. Our results indicated that the high concentrations of AZM maintained in the ISF and skin are related to its potent antibiotic effects, whereas prolonged subinhibitory AZM levels at the target site contribute to the risk of AMR emergence. However, it remains unclear whether this characteristic of AZM in the extracellular tissue space is common among macrolides or specific to AZM alone. Although the fact that macrolides are concentrated in the intracellular space is well known, the kinetics of macrolides in the ISF are not well understood. Further information regarding the distribution of macrolides in the extracellular tissue space could provide new insight into a possible PK-PD-based dosing strategy.

In this study, to compare the pharmacokinetic characteristics of macrolides in the extravascular space, we determined total concentrations of erythromycin (EM) and clarithromycin (CAM), which are 14-membered ring macrolide antibiotic agents, in the ISF of the skin, by using a dissolving microneedle array at the primary infection site (the lung and skin). The obtained results were compared with the previously reported results on AZM.

## 2. Results

### 2.1. Pharmacokinetic Analysis of Macrolides

**Figure 1:**
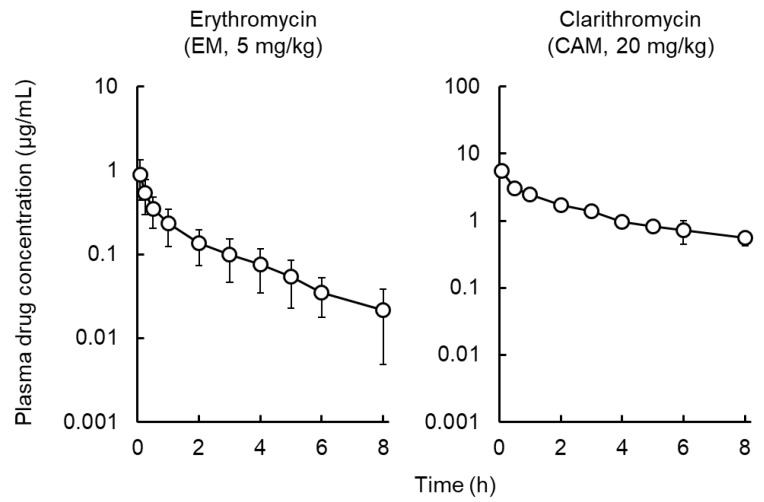
Mean plasma concentration profiles of erythromycin (EM) and clarithromycin (CAM) after intravenous administration of EM (5 mg/kg) or CAM (20 mg/kg) to rats. Each symbol with a bar represents the mean ± S.D. for five rats.

Figure 1 shows the plasma concentration–time profiles of EM and CAM after intravenous (IV) administration of EM or CAM to rats. Table 1 summaries the pharmacokinetic parameters. The obtained results were compared with the previously reported results on AZM. We observed a shorter t_1/2, z_ for EM and CAM than for AZM. The plasma concentrations of EM and CAM at 24 h after administration were not detectable, whereas AZM was detected at 72 h after administration [12]. The Vd values of EM and CAM were lower than that for AZM.

### 2.2. Distribution of Macrolides to Tissues and ISF

**Figure 2:**
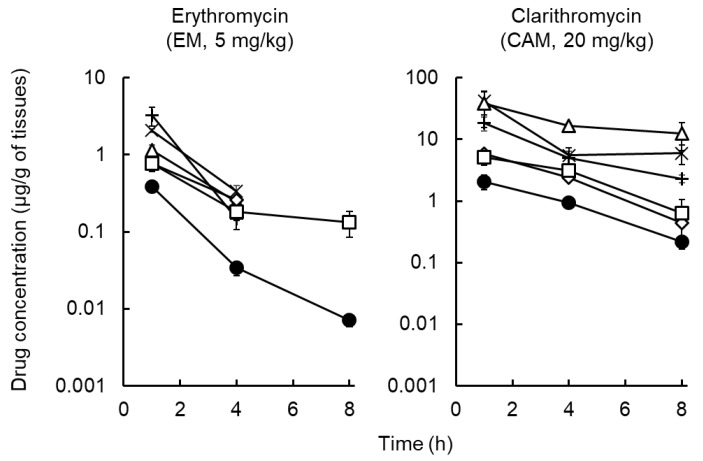
Concentration profiles of erythromycin (EM) and clarithromycin (CAM) in the plasma, liver, kidneys, lung, muscle, and skin after intravenous administration of EM (5 mg/kg) or CAM (20 mg/kg) to rats. (●) plasma; (+) liver; (×) kidneys; (△) lung; (◊) muscle; (□) skin. Each symbol with a bar represents the mean ± S.D. for four to six rats. The EM concentration in the lung, liver, kidney, and muscle at 8 h after administration of EM included 1 or more data of not detected.

Figure 2 depicts the tissue distribution of EM and CAM after IV drug administration. In our preliminary experiment, the plasma concentration of EM and CAM at 24 h was below the limit of quantification (0.001 μg/mL for EM; 0.01 μg/mL for CAM); therefore, we measured the plasma drug levels until 8 h after dosing. The drug concentration in the tissue rapidly reached a maximum value. The distribution of the three macrolides was concentrated in the organs and peripheral tissues including the lung, liver, kidney, muscle, and skin. The drug concentrations in the tissues at each sampling time were much higher (EM: 2.0 to 10.3, CAM: 2.0 to 62.0, AZM: 4.3 to 1082.5 times [12]) than that in the plasma, whereas EM in the lung, liver, kidney and muscle was rapidly eliminated and under the limit of quantification (0.01 μg/g of tissues) at 8 h after administration to rats.

**Figure 3:**
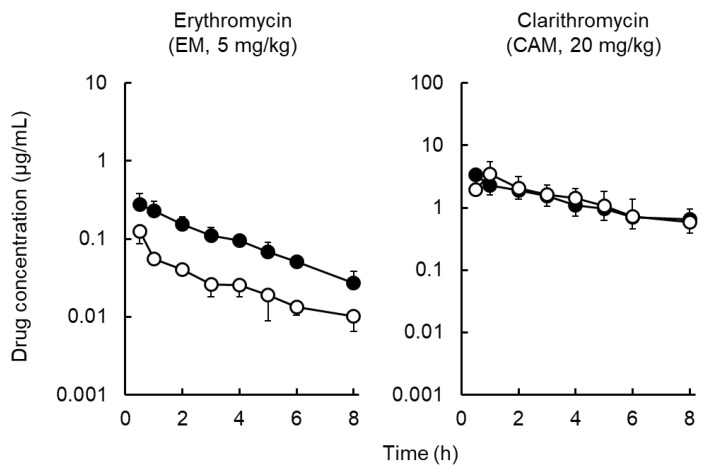
Total concentration profiles of erythromycin (EM) and clarithromycin (CAM) in plasma and interstitial fluid (ISF) after intravenous administration of EM (5 mg/kg) or CAM (20 mg/kg) to rats. (●) plasma; (○) ISF. Each symbol with a bar represents the mean ± S.D. for four to six rats.

Figure 3 depicts the total concentration profiles of EM and CAM in the plasma and ISF after IV administration of each of these substances to rats. The EM concentration in the ISF was 2.1–4.2 times lower than that in the plasma and transiently decreased, whereas the AZM concentration in the ISF was higher than that in the plasma [13]. The CAM concentration in the ISF reached a maximum level at 1 h after administration, indicating that the increase in CAM concentration in the ISF was relatively slow compared to EM. The CAM concentration in the ISF reached the same level as that in the plasma and then transiently decreased.

**Figure 4:**
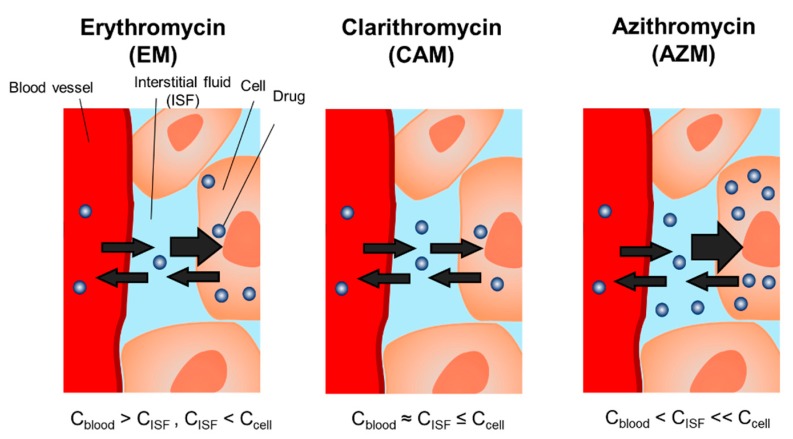
Schematic of transport of three macrolides from blood circulation into extravascular space. C_blood_, drug concentration in blood; C_ISF_, drug concentration in the interstitial fluid; C_cell_, drug concentration in the cell.

Table 2 shows the ISF-plasma and skin-plasma concentration ratios. The order of the ISF/plasma concentration ratios was as follows: AZM (3.8 to 4.9) > CAM (1.2 to 1.5) > EM (0.27 to 0.39), indicating that AZM level in the ISF was higher than that in the plasma; CAM level in the ISF was comparable to that in the plasma; and the EM level in the ISF was lower than that in the plasma. No time-dependent increase in the ISF-plasma concentration ratio was observed for any of the three macrolides. This result indicated that the rate of elimination of macrolides from the ISF was comparable to that from the plasma. On the contrary, the skin–plasma concentration ratio of EM and AZM was significantly and transiently increased in a time-dependent manner; however, for CAM, there was no time-dependent increase in the skin/plasma concentration ratio. This finding indicates that the rate of elimination of EM and AZM from the skin was lower than that from the plasma, resulting in drug retention in the skin. A schematic of transport of three macrolides from blood circulation into extravascular space is illustrated in Figure 4.

## 3. Discussion

This study compared the characteristics of macrolide transport into the ISF by directly collecting ISF samples from a pore formed on the skin by a dissolving microneedle array. Interestingly, the total macrolide level in the ISF was significantly different among EM, CAM, and AZM, whereas higher drug levels in the skin tissue than in the plasma were confirmed for all three macrolides.

A previous clinical study investigating AZM concentrations in the ISF of muscle and subcutaneous fat tissue by microdialysis revealed lower drug levels in the ISF than in the plasma [9,10], in contrast to our results. However, the drug concentrations in the ISF obtained by microdialysis represent protein-unbound drug concentrations due to perfusion. The current study was designed to determine total drug concentrations in the ISF of the skin. Our results indicate that the high total drug concentration in the ISF was specific for AZM, but not EM and CAM.

This AZM-specific characteristic concentration in the ISF is biologically plausible. Phagocyte-driven delivery of macrolides from blood to the extracellular tissue space is one of the important distribution pathways of drugs to infection sites [6,13,14]. A potential mechanism for phagocyte uptake of macrolides is ion-trapping, in which basic compounds such as macrolides are protonated and concentrated in acidic organelle compartments after the drugs are transported into the cell [4,6,7,14]. The transportability of AZM into human polymorphonuclear neutrophils or animal macrophages is markedly higher than that of other macrolides [6,14,15,16]. AZM has a diprotic base molecular structure [17], which could promote greater accumulation of AZM in phagocytic cells, compared with CAM or EM. These results suggest that the differences in the macrolide accumulation in phagocytic cells in the ISF result in the total drug concentration in the ISF and its antimicrobial activity at the primary infection site. To estimate antimicrobial effects and develop PK-PD-based proper dosing strategy, total concentrations of macrolides in the ISF should be considered. The monitoring of plasma concentrations of macrolides may alternatively estimate the total drug level in the ISF, because the elimination rate constant of the drug from ISF was comparable to that from the plasma for all three macrolides.

EM levels in the ISF were lower than in the plasma and skin tissues, whereas EM retention was observed in only the skin tissue, not other organ/tissues. These observations indicated that EM can be well distributed into the cells of skin tissue and the slow back-distribution of EM from intracellular to extracellular compartments would occur. These results also suggest that a specific binding protein to EM may exist in the cells of skin. In vitro antibacterial activity against gram-positive bacteria, including *Staphylococcus epidermidis*, *Staphylococcus aureus*, and *Streptococcus pyogenes*, is comparable among the three macrolides, and all three are used for the treatments for these infections [18]. After infection, *S. epidermidis* is present mainly in the extracellular tissue space, but *S. aureus* and *S. pyogenes* can invade nonphagocytic cells [19,20]. A recent clinical study reported that *S. epidermidis* isolated from patients with coagulase-negative staphylococci bacteremia exhibited a high rate of resistance against EM (87.7%) [21]. Taken together, these results suggest that the subinhibitory EM levels in the ISF, which is the target site for *S. epidermidis*, may favor the emergence of bacterial resistance. Therapeutic dose selection considering both the kinetic characteristics of macrolides in the ISF and the location of target strains can help prevent the emergence of bacterial resistance.

The clinical efficacy of AZM is better in treating community-acquired pneumonia with macrolide-resistant *Streptococcus pneumoniae* [22,23]. Although this in vivo-in vitro paradox may be due to AZM-phagocyte-driven delivery to inflamed sites and an inhibitory effect on pneumolysin production [22], the details remain poorly understood. Our findings lead us to speculate that AZM-specific high total concentrations in the ISF and intracellular tissues may contribute to better clinical efficacy against macrolide-resistant *S. pneumoniae*. Further studies are needed to elucidate the contribution of drug levels and retention in the ISF to antimicrobial effects on macrolide-resistant strains and the emergence of bacterial resistance.

Several limitations of this study should be acknowledged. First, the macrolide concentrations in the ISF were examined and compared in healthy rats, not in infection model rats. It was previously reported that AZM exposure in ISF in the infected and inflammatory tissues was increased compared to noninflammatory tissues [24,25]. There is a possibility that inflammation may enhance the vascular permeability of phagocytes and phagocyte-driven delivery of macrolides. Moreover, inflammation decreases the pH value in the extravascular space [26], which may lead to increasing AZM ionization in the ISF. This further increase of ionized-AZM would result in AZM stasis in the ISF because of lower permeability of the ionized form into tissue cells than the un-ionized form. Under inflammation conditions, the pharmacokinetic characteristics of drugs in the ISF may be changed. To confirm the macrolide distribution in the ISF at inflammation sites, further studies are needed. Second, it was difficult to assess the ISF protein-binding ratio and the drug concentrations in phagocytic cells due to limited ISF sample volumes from the skin. Thus, the detailed distribution of macrolides in the ISF compartment could not investigated. In general, the protein binding is important for efficacy of drugs. Although we could not evaluate the protein binding ratio, the current results found the differences of localization of macrolides in the skin tissue compartment and drug kinetics in the ISF compartment. The current findings can contribute to develop the therapeutic dose selection method and an appropriate dosing strategy based on the PK-PD theory considering the kinetics of drugs in ISF. Finally, there is the difficulty in applying the current results of animal studies directly toward developing an appropriate clinical dosing strategy. To determine the impact of pharmacokinetic characteristics in the ISF on clinical efficacy and the emergence of bacterial resistance, additional clinical studies are needed.

## 4. Materials and Methods

### 4.1. Materials

EM and CAM were obtained from Wako Pure Chemical Industries Ltd. (Osaka, Japan). DMN array chips were kindly provided by BioSerenTach Inc. (Kyoto, Japan). All other reagents were of analytical grade and were used without further purification.

### 4.2. Pharmacokinetic Study of Macrolides

All animal studies were approved by an institutional review board (Permit number: PKPD-15-001, Date of approval: 27 April 2015) and performed in accordance with the Kyoto Pharmaceutical University Guidelines for Animal Experimentation. Male Wistar rats (10 weeks of age) were purchased from Nippon SLC Co., Ltd. (Hamamatsu, Japan). All rats were housed in a temperature-controlled facility with free access to food and water prior to the studies.

To investigate the pharmacokinetics of macrolides and compare our findings with the previous results for AZM, a time-course of the plasma concentrations of EM and CAM was evaluated. After intraperitoneal administration of a mixture of 0.375 mg/kg medetomidine, 2.0 mg/kg midazolam, and 2.5 mg/kg butorphanol anesthesia, 5 mg/kg EM (5 mg/mL in 10% propylene glycol-methanol 1:1 *v*/*v* solution) or 20 mg/kg CAM (20 mg/mL in 1% phosphate solution), was IV administered to rats. Although macrolides are generally administered orally, IV administration were selected to exclude variations in the drug absorption process and to maintain continuity allowing comparison of the results of our previous and current study. The dosage of EM and CAM was determined based previous animal studies [27,28,29] and clinical dosages. Blood (250 μL) was withdrawn into heparinized centrifuge tubes from the external left jugular vein at 5, 15, and 30 min, as well as 1, 2, 3, 4, 5, 6, 8, and 24 h after drug administration. The blood samples were centrifuged at 14,000 × g for 15 min, and the collected plasma samples were stored at −80 °C for analysis.

### 4.3. Tissue Distribution of Macrolides

To investigate the distribution of macrolides in tissues, the concentrations of EM and CAM in the lung, liver, kidneys, muscle, and skin of rats were determined according to previously reported procedures [12]. At 1, 4, and 8 h after IV administration of 5 mg/kg EM or 20 mg/kg CAM, the rats were euthanized by exsanguination. The sampling schedule was determined based on our previous study [12,29] and the lower limit of quantification. Tissues were perfused with phosphate-buffered saline (PBS, pH 7.4) to remove blood. After the lung, liver, kidneys, muscle, and abdominal skin (with the hair removed) were collected and washed with PBS, they were homogenized in PBS (nine-fold volumes of each sample weight) using a homogenizer (PT 10-35 GT; Kinematica AG, Lucerne, Switzerland). After centrifugation of the homogenate sample at 3000 × g for 15 min, the supernatant fractions were stored at −80 °C until analysis.

### 4.4. Distribution of Macrolides to ISF

To investigate the distribution of macrolides in the ISF, ISF samples were collected from the abdominal skin of rats (with the hair removed) according to previously reported methods [10,11,12]. After IV administration of 5 mg/kg EM or 20 mg/kg CAM, 5 μL of ISF was collected from a pore on the abdominal skin formed by the DMN array chip with an applicator at 0.5, 1, 2, 3, 4, 5, 6, and 8 h after IV administration of drugs. In order to evaluate the distribution of drugs from the plasma to the ISF, 0.25 mL blood samples were also collected from the left jugular vein at the same time points. After centrifugation of the blood samples in heparinized tubes at 14,000 × g for 15 min, the collected ISF and plasma samples were stored at −80 °C for analysis.

### 4.5. Macrolides Assay

The assay for EM or CAM was conducted using liquid chromatography-tandem mass spectrometry (LC-MS/MS), according to a previously-reported method with minor modifications [30,31]. Briefly, 5 μL of ISF was diluted with 95 μL of distilled water. For the EM assay, 150 μL of acetonitrile was added to a 100 μL-aliquot of a plasma, tissue homogenate, or ISF sample. After vigorous mixing for 15 s, the mixture was centrifuged for 15 min at 14,000 × g. The obtained supernatant (30 µL) was injected into the LC-MS/MS system. For the CAM assay, 0.02 M sodium carbonate solution (300 μL) was added to a 100 μL-aliquot of a plasma, tissue homogenate, or ISF sample containing 10 μL internal standard (AZM solution, 1 μg/mL in 50% methanol). After mixing vigorously for 15 s, a mixture of acetic acid and isopropyl alcohol (1.5 mL; 95:5, *v*/*v*) was added. After vortexing for over 30 s and centrifugation for 15 min at 14,000 × g, the supernatant was transferred to a fresh centrifuge tube and then evaporated to dryness under a stream of nitrogen at 60 °C. Then, 100 μL 10 mM ammonium acetate and acetic acid (35:64.5:0.5, *v*/*v*/*v*) was added to the resulting residue and vortexed for over 30 s. The resulting reconstituted solution (30 µL) was injected into the LC–MS/MS system. The lower limit of quantification (LLOQ) for the analytes was described as follow; EM in plasma and ISF: < 0.001 μg/mL; EM in tissue sample: < 0.01 μg/g of tissues; CAM in plasma and ISF: < 0.01 μg/mL; CAM in tissue sample: < 0.1 μg/g of tissues.

The LC–MS/MS system consisted of an API 3200 triple-quadrupole mass spectrometer (Applied Biosystems/MDS Sciex, Foster City, CA, USA). The mobile phase for EM was 5 mM ammonium acetate and acetic acid (1:1, *v*/*v*). The flow rate of the mobile phase was 0.2 mL/min, and chromatographic separations were conducted using a Quicksorb ODS (2.1 × 150 mm, 5 μm size; Chemco Scientific Co., Ltd., Osaka, Japan), maintained at 50 °C. The mass spectrometer used a selected reaction monitoring method in positive ion mode, with 734.3→158 for EM. The analytical conditions for CAM and the internal standard AZM were previously reported [12].

### 4.6. Pharmacokinetic Analysis

To obtain pharmacokinetic parameters for macrolides, non-compartmental pharmacokinetic analysis was conducted using WinNonlin^®^ Version 6.3 software (Certara USA, Inc., Princeton, NJ, USA). The area under the plasma concentration–time curve from time of dosing to infinity (AUC_0–∞_) and that under the first moment curve to the last measured plasma concentration from time of dosing to infinity (AUMC_0–∞_) were determined by the linear trapezoidal rule. The terminal slope (λ_z_) was calculated by linear regression using at least three data points from the terminal portion of the plasma concentration–time curve using the Best Fit program in WinNonlin^®^. The half-life (*t*_1/2, z_) was calculated by the formula *t*_1/2, z_ = ln2/λ_z_. The mean residence time (MRT) was calculated using the formula AUMC_0–∞_/AUC_0–∞_. Total plasma clearance (CL_tot_) was calculated using the formula D/AUC_0–∞_; where, D is the administered dose of macrolides. The distribution volume (Vd) was calculated by multiplying CL_tot_ by MRT.

### 4.7. Statistical Analysis

All values are shown as the mean ± standard deviation (S.D.). Comparisons across multiple groups were made using one-way analysis of variance (ANOVA) followed by Tukey’s test. The differences between the means were considered statistically significant when *p* < 0.05. To evaluate the distribution of macrolides from plasma to the ISF or tissues, the drug concentration ratio to plasma was calculated for individual rats.

## 5. Conclusions

We found that the high total drug exposure in the ISF is specific for AZM among the three macrolides investigated, which was demonstrated by directly collecting ISF samples. The differences in macrolide accumulation in phagocytic cells may impact the total concentration of macrolides in the ISF. Lower total EM levels in the ISF than in the plasma were found, which would be expected to contribute to the emergence of bacterial resistance. The pharmacokinetic characteristics of macrolides in the ISF contribute to both the pharmacological effects and the emergence of bacterial resistance at the primary infection site. Our results can help in the development of PK-PD-based antimicrobial chemotherapeutic strategies using macrolides. Moreover, our findings may have important implications for individualized dose selection of macrolides considering the infecting bacterium. However, further studies are required to better understand the relationship among the differences in macrolide distribution characteristics in the ISF, their antibacterial efficacy, and macrolide resistance.

## Figures and Tables

**Table 1:** Pharmacokinetic parameters after intravenous administration of erythromycin (5 mg/kg) or clarithromycin (20 mg/kg) to rats, and comparison with previously reported results on azithromycin (10 mg/kg).

Pharmacokinetic Parameters	Erythromycin (EM)	Clarithromycin (CAM)	Azithromycin ^a)^ (AZM)
*t*_1/2, z_ (h)	1.8 ± 0.4	3.3 ± 0.8	53.5 ± 32.6
*AUC*_0–∞_ (μg∙h/mL)	1.1 ± 0.5	13.8 ± 2.6	10.2 ± 4.1
*CL*_tot_ (L/h/kg)	5.6 ± 2.6	1.5 ± 0.3	1.2 ± 0.7
*Vd* (L/kg)	13.8 ± 5.1	6.8 ± 0.8	68.5 ± 30.2
*MRT* (h)	2.2 ± 0.5	4.3 ± 1.0	64.9 ± 47.0

Each value represents the mean ± S.D. of five rats. *t*_1/2, z_, elimination half-life; AUC_0–∞_, area under the plasma concentration–time curve from time of dosing to infinity; CL_tot_, total plasma clearance; Vd, distribution volume; MRT, mean residence time. ^a)^ These results were previously reported in [12].

**Table 2:** Pharmacokinetic parameters after intravenous administration of erythromycin (5 mg/kg) and clarithromycin (20 mg/kg) to rats, and comparison with previously reported results on azithromycin (10 mg/kg).

Time (h)	Erythromycin (EM)		Clarithromycin (CAM)		Azithromycin ^a)^ (AZM)	
	ISF/Plasma	Skin/Plasma	ISF/Plasma	Skin/Plasma	ISF/Plasma	Skin/Plasma
1	0.27 ± 0.10	3.2 ± 0.9	1.5 ± 0.9	2.5 ± 0.4	3.8 ± 1.6	4.3 ± 1.5
4	0.27 ± 0.05	6.3 ± 2.3	1.5 ± 0.7	3.4 ± 0.4	5.0 ± 1.3	36.3 ± 23.8
8	0.39 ± 0.08	11.9 ± 3.8 *^, #^	1.2 ± 0.9	2.9 ± 1.8	–	–
72	–	–	–	–	4.9 ± 0.8	83.1 ± 76.2 *

Interstitial fluid, ISF. This ratio was determined by comparing the drug concentration in the tissue with that in plasma of individual rats. Each value represents the mean ± S.D. of four to seven rats. Statistical significance was evaluated by one-way analysis of variance (one-way ANOVA) with post-hoc comparisons by the Tukey’s test. * *p* < 0.05 statistically significant difference vs. the data at 1 h. ^#^ *p* < 0.05 statistically significant difference vs. the data at 4 h. ^a)^ These results were previously reported in [12].
